# COVID-19 Disease Map, building a computational repository of SARS-CoV-2 virus-host interaction mechanisms

**DOI:** 10.1038/s41597-020-0477-8

**Published:** 2020-05-05

**Authors:** Marek Ostaszewski, Alexander Mazein, Marc E. Gillespie, Inna Kuperstein, Anna Niarakis, Henning Hermjakob, Alexander R. Pico, Egon L. Willighagen, Chris T. Evelo, Jan Hasenauer, Falk Schreiber, Andreas Dräger, Emek Demir, Olaf Wolkenhauer, Laura I. Furlong, Emmanuel Barillot, Joaquin Dopazo, Aurelio Orta-Resendiz, Francesco Messina, Alfonso Valencia, Akira Funahashi, Hiroaki Kitano, Charles Auffray, Rudi Balling, Reinhard Schneider

**Affiliations:** 1 Luxembourg Centre for Systems Biomedicine, University of Luxembourg, Belvaux, Luxembourg; 2 Ontario Institute for Cancer Research, Toronto, Canada; 3 College of Pharmacy and Health Sciences, St. John’s University, Queens, NY USA; 4 Institut Curie, PSL Research University, Mines Paris Tech, Inserm, Paris, France; 5 Department of Biology, Univ. Évry, University of Paris-Saclay, Genopole, 91025 Évry, France; 6 European Molecular Biology Laboratory, European Bioinformatics Institute (EMBL-EBI), Hinxton, UK; 7 Institute of Data Science and Biotechnology, Gladstone Institutes, San Francisco, United States; 8 Department of Bioinformatics-BiGCaT, NUTRIM, Maastricht University, Maastricht, The Netherlands; 9 Maastricht Centre for Systems Biology, Maastricht University, Maastricht, The Netherlands; 10 Helmholtz Zentrum München, Institute of Computational Biology, Neuherberg, Germany; 11 Center for Mathematics, Technische Universität München, Garching, Germany; 12 Faculty of Mathematics and Natural Sciences, University of Bonn, Bonn, Germany; 13 University of Konstanz, Department of Computer and Information Science, Konstanz, Germany; 14 Monash University, Faculty of Information Technology, Melbourne, Australia; 15 Computational Systems Biology of Infection and Antimicrobial-Resistant Pathogens, Institute for Bioinformatics and Medical Informatics (IBMI), University of Tübingen, 72076 Tübingen, Germany; 16 Department of Computer Science, University of Tübingen, 72076 Tübingen, Germany; 17 German Center for Infection Research (DZIF), partner site, Tübingen, Germany; 18 Department of Molecular and Medical Genetics, School of Medicine, Oregon Health & Science University, Portland, USA; 19 Department of Systems Biology & Bioinformatics, University of Rostock, Rostock, Germany; 20 Stellenbosch Institute of Advanced Study (STIAS), Wallenberg Research Centre at Stellenbosch University, 7602 Stellenbosch, South Africa; 21 Research Programme on Biomedical Informatics, Hospital del Mar Medical Research Institute, Department of Experimental and Health Sciences, Pompeu Fabra University, Barcelona, Spain; 22 Clinical Bioinformatics Area, Fundación Progreso y Salud. Hosp. Virgen del Rocío, Sevilla, Spain; 23 Bioinformatics in Rare Diseases. Centro de Investigación Biomédica en Red de Enfermedades Raras, Fundación Progreso y Salud, Hosp. Virgen del Rocío, Sevilla, Spain; 24 INB-ELIXIR-es, FPS, Hospital Virgen del Rocío, Sevilla, 42013 Spain; 25 Institute of Biomedicine of Seville (IBIS), Hospital Virgen del Rocio, 41013 Sevilla, Spain; 26 HIV, Inflammation and Persistence Unit, Virology Department, Institut Pasteur, Paris, France; 27 Dipartimento di Epidemiologia Ricerca Pre-Clinica e Diagnostica Avanzata, National Institute for Infectious Diseases “Lazzaro Spallanzani” I.R.C.C.S., Rome, Italy; 28 COVID 19 INMI Network Medicine for IDs Study Group, National Institute for Infectious Diseases “Lazzaro Spallanzani” I.R.C.C.S., Rome, Italy; 29 Barcelona Supercomputer Center (BSC), Barcelona, Spain; 30 Institucio Catalana de Recerca I Estudis Avançats (ICREA), Barcelona, Spain; 31 Department of Biosciences and Informatics, Keio University, Yokohama, Kanagawa Japan; 32 The Systems Biology Institute, Shinagawa, Tokyo Japan; 33 Okinawa Institute of Science and Technology Graduate University, Kunigami, Okinawa Japan; 34 Sony Computer Science Laboratories, Inc., Tokyo, Japan; 35 European Institute for Systems Biology and Medicine (EISBM), Vourles, France; 36 Bio Sorbonne Paris Cité, Université de Paris, Paris, France

**Keywords:** Gene regulatory networks, Cellular signalling networks, Biochemical reaction networks, Computational models

## Abstract

Researchers around the world join forces to reconstruct the molecular processes of the virus-host interactions aiming to combat the cause of the ongoing pandemic.

We announce the COVID-19 Disease Map (10.17881/covid19-disease-map), an effort to build a comprehensive, standardized knowledge repository of SARS-CoV-2 virus-host interaction mechanisms, guided by input from domain experts and based on published work. This knowledge, available in the vast body of existing literature^1,2^ and the fast-growing number of new SARS-CoV-2 publications, needs rigorous and efficient organization in both human and machine-readable formats.

This endeavour is an open collaboration between clinical researchers, life scientists, pathway curators, computational biologists and data scientists. Currently, 162 contributors from 25 countries around the world are participating in the project, including partners from Reactome^3^, WikiPathways^4^, IMEx Consortium^5^, Pathway Commons^6^, DisGeNET^7^, ELIXIR^8^, and the Disease Maps Community^9^. With this effort, we aim for long-term community-based development of high-quality models and knowledge bases, linked to data repositories.

The COVID-19 Disease Map will be a platform for visual exploration and computational analyses of molecular processes involved in SARS-CoV-2 entry, replication, and host-pathogen interactions, as well as immune response, host cell recovery and repair mechanisms. The map will support the research community and improve our understanding of this disease to facilitate the development of efficient diagnostics and therapies. Figure 1 illustrates the initial scope and layout of the map and its life cycle.

**Fig. 1:**
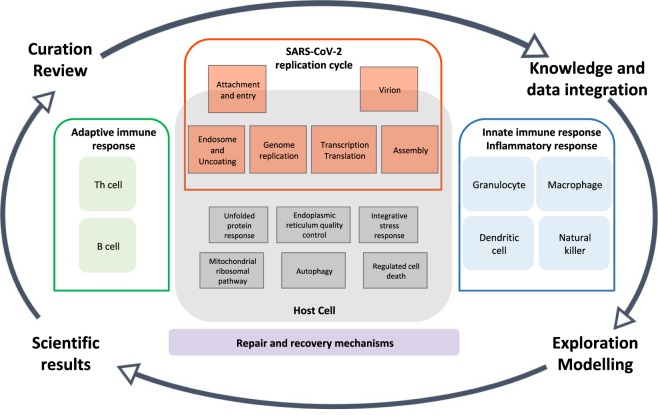
The overview of the COVID-19 Disease Map project. The map focuses on SARS-CoV-2 replication cycle, its interactions with the host, reaction of the immune system and repair mechanisms. The curated and reviewed content will be continuously integrated and cross-linked with data and knowledge bases, to support visual and computational exploration, as well as disease modelling efforts. The acquired results will benefit the research community and provide feedback to refine the scope of curation activities.

At the time this Comment went to press, the COVID-19 Disease Map contains pathways of (i) the virus replication cycle and its transcription mechanisms; (ii) SARS-CoV-2 impact on ACE2-regulated pulmonary blood pressure, apoptosis, Cul2-mediated ubiquitination, heme catabolism, Interferon 2 and PAMP signalling, and endoplasmic reticulum stress; (iii) SARS-CoV-2 proteins Nsp4, Nsp6, Nsp14 and Orf3a. Moreover, the map incorporates the COVID-19 collection of WikiPathway diagrams^10^ and a pre-published genome-scale metabolic model of human alveolar macrophages with SARS-CoV-2^11^. All these contributed open-access resources are referenced at https://fairdomhub.org/projects/190#models.

By combining diagrammatic representation of COVID-19 mechanisms with underlying models, the map fulfils a dual role. First, it is a graphical, interactive representation of disease-relevant molecular mechanisms linking different knowledge bases. Second, it is a computational resource of reviewed content for graph-based analyses^12^ and disease modelling^13^. Thus, it provides a platform for domain experts, such as clinicians, virologists, and immunologists, to collaborate with data scientists and computational biologists for a rigorous model building, accurate data interpretation and drug repositioning. It offers a shared mental map to understand gender, age, and other susceptibility features of the host, disease progression, defence mechanisms, and response to treatment. Finally, it can be used together with the maps of other human diseases to study comorbidities.

In the construction of the COVID-19 Disease Map, we rely on multiple tools for curation and review the contributed content in a distributed, on-the-fly manner. Most importantly, already at this early stage, we involve practising physicians and clinical researchers to improve the scope and quality of the map. Motivated by our curation experience and the number of participants contributing to the construction of the map, we propose and regularly revise common curation guidelines and follow commonly-accepted exchange standards. Moreover, given the multicellular and multiorgan nature of COVID-19 infection and the complexity of the underlying molecular mechanisms, we envisage the map as a hierarchical structure of interconnected functional modules. We anticipate that the structure of the map will evolve as new knowledge about the disease is revealed.

This distributed, multi-tool, multi-group approach is dictated by the urgency of the ongoing pandemic, by the high volume of new COVID-19-related publications, and by an impressive response from the research community. In this challenging situation, it is imperative that community-based approaches are used to develop high-quality models and data. To ensure a transparent view of the contributors and community resources, we rely on the support of FAIRDOMHub^14^. All data and curation guidelines related to the COVID-19 Disease Map are available at https://fairdomhub.org/projects/190.

We invite curators to join the project and contribute to building a solid foundation of COVID-19 molecular and cellular mechanisms using systems biology standards^15–17^. Moreover, we request support from domain experts to advise on the content and to review the map, improving its quality and applicability, as well as experts in modelling to accelerate the development of efficient diagnoses, treatments, and vaccines in response to the ongoing pandemic.
